# Supplementary material for: A diverse host thrombospondin-type-1 repeat protein repertoire promotes symbiont colonization during establishment of cnidarian-dinoflagellate symbiosis
Source: eLife. 2017 May 8;6:e24494. doi: 10.7554/eLife.24494 (PMC5446238; doi:10.7554/eLife.24494)
Supplement: Supplementary file 2. — DOI: http://dx.doi.org/10.7554/eLife.24494.024 [file elife-24494-supp2.docx]

Supplementary file 2: Summary of fluorescent dyes and their excitation and emission wavelengths used for confocal microscopy

| Labelled structure, activity or molecule | Dye | Excitation wavelength | Emission wavelength |
| --- | --- | --- | --- |
| Host and symbiont nuclei | 1.5μM DAPI | 405nm | 420-480nm |
| Dinoflagellates | None (detection of autofluorescence) | 543nm | 600-700nm |
| Host symbiosome plasma membranes | DilC lipophilic membrane stain (Molecular probes) | 549nm | 565nm |
| TSR domain containing proteins | Alexa fluor546 goat anti-rabbit secondary antibody (Molecular probes) | 556nm | 573nm |
