# Supplementary material for: A diverse host thrombospondin-type-1 repeat protein repertoire promotes symbiont colonization during establishment of cnidarian-dinoflagellate symbiosis
Source: eLife. 2017 May 8;6:e24494. doi: 10.7554/eLife.24494 (PMC5446238; doi:10.7554/eLife.24494)
Supplement: Supplementary file 3. — DOI: http://dx.doi.org/10.7554/eLife.24494.025 [file elife-24494-supp3.docx]

Supplementary file 3. Primers for initial PCR of TSR sequences.

| Gene | Primer Names | Primer sequence |
| --- | --- | --- |
| Ap_Sema5 | SemaF260  SemaR769 | 5’-GTC ACT TGC TGG TTC TTG CA-3’  5’-CTT CCA AGT ACA CTG CGG GT-3’ |
|  | SemaF608  SemaR1350 | 5’-ACA CGA GTG ATG AAT TGG TCT T-3’  5’-AAG GAG TAC GCA CAA ATG GC-3’ |
|  | SemaF1207  SemaR1983 | 5’-AGA TTG TTC TAC ACC AGG CAC-3’  5’-TGC CAA CCG TGA TCC CTA TT-3’ |
|  | SemaF1841  SemaR2639 | 5’-ACC CCG AAT AAG CTG GTG AA-3’  5’-GTT TTC TGG ACT GCA TGC CA-3’ |
|  | SemaF2555  SemaR3367 | 5’-ATC CAA CCA ACA ACA CCA CC-3’  5’-CCA CTG ACC ACA TAT ACC GC-3’ |
| Ap_Trypsin-like | TrypsinF53  TrypsinR841 | 5’-CAG TTT AAC GCG CGT CAT TC-3’  5’-ACT CCA CTA CCA CAC GTC AC-3’ |
|  | TrypsinF740  TrypsinR1533 | 5’-GTA CAC CAC CGC GTG TAA TC-3’  5’-TGC ATA CTG GTT TGA CGT GC-3’ |
|  | TrypsinF861  TrypsinR1769 | 5’-TGT GAC AGC CCT CCT CCT AA-3’  5’-AAA TGG TCC ACC GCT GTC TC-3’ |
|  | TrypsinF1420  TrypsinR2122 | 5’-CAC AAG TTT GCA TGC ATC CC-3’  5’-TAT TGC TCC TCG GCC AGA TC-3’ |
