# Supplementary material for: A diverse host thrombospondin-type-1 repeat protein repertoire promotes symbiont colonization during establishment of cnidarian-dinoflagellate symbiosis
Source: eLife. 2017 May 8;6:e24494. doi: 10.7554/eLife.24494 (PMC5446238; doi:10.7554/eLife.24494)
Supplement: Supplementary file 4. — DOI: http://dx.doi.org/10.7554/eLife.24494.026 [file elife-24494-supp4.docx]

**Supplementary file 4.** Primers used for qPCR of Ap_Sema5 and Ap_Trypsin-like amplicons.

| **Gene** | **Primer Names** | **Primer sequence** |
| --- | --- | --- |
| Ap_Sema5 | SemaF2612 qPCR  SemaR2766 qPCR | 5’- TCG ATG CCC GAA CGA CCG AG-3’  5’- CTC CGG TCA CGA TAG CTG CTC C-3’ |
| Ap_Trypsin-like | TrypsinF1105 qPCR  TrypsinR1259 qPCR | 5’-GCC ATG GCA AGC CGG CTT AA-3’  5’-CGC GTA GTC GAG GCT CAG GT-3’ |
