# Supplementary material for: A diverse host thrombospondin-type-1 repeat protein repertoire promotes symbiont colonization during establishment of cnidarian-dinoflagellate symbiosis
Source: eLife. 2017 May 8;6:e24494. doi: 10.7554/eLife.24494 (PMC5446238; doi:10.7554/eLife.24494)
Supplement: Source code 1. — DOI: http://dx.doi.org/10.7554/eLife.24494.027 [file elife-24494-code1.htm]

Statistical analyses


Code 

- Show All Code
- Hide All Code
- Download Rmd

# Statistical analyses


# 1. TSP antibody experiment

## 1.1 TSP antibody model


```
antiTSP_mod_data <- read.csv('Figure 5A source data1 model.csv', stringsAsFactors = F) %>% 
  mutate(Infection = infection+0.01,
       anemone = paste(Anemone,hours,Treatment),
       Hours = as.factor(hours)) 

# run mixed effects model - nlme for contrast methods
antiTSP_model_test <- lme(log(Infection)~ Hours*Treatment,
                          random=~1|anemone,
                          data=antiTSP_mod_data)

plot(antiTSP_model_test)
```


```
sm_test <- summary(antiTSP_model_test)
anova(antiTSP_model_test, type='m')
```

## 1.2 TSP antibody model contrasts at time points

These are contrasts between the FSW and IGG controls and the anti-TSR treatment


```
antiTSP_lsmobj <- lsmeans(antiTSP_model_test, ~ Treatment|Hours)


summary(as.glht(pairs(antiTSP_lsmobj),by=NULL))
confint(pairs(antiTSP_lsmobj))
```

## 1.3 TSP antibody model plot


```
# summary file of means and SE per treatment and time-point
antiTSP <- read.csv('Figure 5A source data2.csv', stringsAsFactors = F)

antiTSP$Treatment[antiTSP$Treatment == 'TSP antibody'] <- 'Thrombospondin antibody'


ggf1 <- ggplot(antiTSP,aes(x=Hours,y=X..Infection,colour = Treatment, shape = Treatment)) + 
  geom_line() + 
  geom_point() +
  geom_pointrange(aes(ymin = X..Infection - se, ymax = X..Infection + se)) +
  geom_text(data=antiTSP,aes(x=Hours, y=X..Infection+se+0.5,label=c(NA,NA,NA,NA,NA,'**',NA,NA,'***',NA,NA,'***',NA,NA,'***')),show.legend = FALSE)+
  #scale_y_continuous(labels = percent)+
  scale_x_continuous(breaks = c(0,24,48,72,96,120))+
  ylab('') +
  scale_colour_manual('Thrombospondin antibody',values = c("#56B4E9","#E69F00",  "#009E73")) +
  scale_shape_discrete('Thrombospondin antibody')+
  theme_cowplot()+ 
  theme(legend.title = element_text(colour = NA),
        axis.title.x = element_text(colour = NA),
        axis.text.x = element_text(colour = NA))
```

# 2. Soluable TSP experiment

## 2.1 Soluable TSP model


```
tsp_soluble_mod_data <- read.csv('Figure 5B source data1 model.csv', stringsAsFactors = F) %>% 
  mutate(Infection = infection+0.01,
         anemone = paste(Anemone,hours,Treatment),
         Hours = as.factor(hours)) 

# run mixed effects model
tsp_soluble_model_test <- lme(log(Infection)~ Hours*Treatment,
                              random=~1|anemone,
                              data=tsp_soluble_mod_data)


plot(tsp_soluble_model_test)
```


```
# summaries
sm_test <- summary(tsp_soluble_model_test)
anova(tsp_soluble_model_test, type='m')
```

## 2.2 Soluable TSP model contrasts


```
tsp_soluble_lsmobj <- lsmeans(tsp_soluble_model_test, ~ Treatment|Hours)

summary(as.glht(pairs(tsp_soluble_lsmobj),by=NULL))
confint(pairs(tsp_soluble_lsmobj),by=NULL)
```

## 2.3 Soluable TSP plot


```
# summary file of means and SE per treatment and time-point
tsp_soluble <- read.csv('Figure 5B source data2.csv', stringsAsFactors = F)

tsp_soluble$Treatment[tsp_soluble$Treatment == 'Hs TSP-1'] <- 'Hs TSR'


ggf2 <- ggplot(tsp_soluble,aes(x=Hours, 
                       y=X..Infection, 
                       colour = Treatment, shape = Treatment)) + 
  geom_line() + 
  geom_point() +
  geom_pointrange(aes(ymin = X..Infection - se, ymax = X..Infection + se)) +
  geom_text(data=tsp_soluble,aes(x=Hours, y=X..Infection+se+c(rep(0.5,5),1.5,rep(0.5,4)),label=c(NA,NA,NA,'***',NA,NA,NA,'**',NA,NA)),show.legend = FALSE)+
  #scale_y_continuous(labels = percent)+
  scale_x_continuous(breaks = c(0,24,48,72,96,120))+
  ylab('% colonisation') +
  scale_colour_manual('Thrombospondin antibody',values = c("#56B4E9","#CC79A7")) +
  scale_shape_discrete('Thrombospondin antibody')+
  theme_cowplot()+ 
  theme(legend.title = element_text(colour = NA),
        axis.title.x = element_text(colour = NA),
        axis.text.x = element_text(colour = NA))
```

# 3 TSR peptide colonisation

## 3.1 TSR peptide colonisation


```
sm_test <- summary(peptide_model_test)
anova(peptide_model_test,type='marginal')
```

## 3.2 Peptide contrasts


```
peptide_lsmobj <- lsmeans(peptide_model_test, ~ Treatment|Hours)

summary(as.glht(pairs(peptide_lsmobj),by=NULL))
confint(pairs(peptide_lsmobj), by=NULL)
```

## 3.3 Plot peptide colonisation


```
peptide_infection <- read.csv('Figure 5C source data2.csv')

ggf3 <- ggplot(peptide_infection,aes(x=Hours,y=X..Infection,colour = Treatment, shape = Treatment)) + 
  geom_line() + 
  geom_point() +
  geom_pointrange(aes(ymin = X..Infection - se, ymax = X..Infection + se)) +
  geom_text(data=peptide_infection,aes(x=Hours, y=X..Infection+se+0.5,label=c(NA,NA,NA,NA,NA,'***',NA,NA,'***',NA,NA,'***',NA,NA,NA)),show.legend = FALSE)+
  geom_text(data=peptide_infection,aes(x=Hours, y=X..Infection-se-c(rep(1,nrow(peptide_infection)-2),0.1,NA),label=c(NA,NA,NA,NA,'***',NA,NA,'***',NA,NA,'***',NA,NA,NA,NA)),nudge_y = -0.2,show.legend = FALSE)+
  #scale_y_continuous(labels = percent)+
  scale_x_continuous(breaks = c(24,48,72,96,120))+
  ylab('') +
  scale_colour_manual('Thrombospondin antibody',values = c("#56B4E9", "#0072B2", "#D55E00")) +
  scale_shape_discrete('Thrombospondin antibody')+
  theme_cowplot()+ 
  theme(legend.title = element_text(colour = NA))
```

# 4. Plots


```
all_figures <- cowplot::plot_grid(ggf1,ggf2,ggf3,ncol=1)
all_figures
ggsave(all_figures,filename = 'Figure 5.pdf',width = 6,height=8)
```

# 5. qPCR analysis


```
qPCR <- read.csv('Figure 6 source data.csv',
                 stringsAsFactors = F)

qPCR$Time <- as.factor(qPCR$Time)
qPCR$Symbiont <- as.factor(qPCR$Symbiont)
```


## 5.1 Ap\_Sema5 analysis


```
Semamod<-lm(Sema~oral_disc+Symbiont*Time, data = qPCR)
anova(Semamod)

Semamod_lsmobj <- lsmeans(Semamod, ~ Symbiont|Time)
summary(as.glht(pairs(Semamod_lsmobj),by=NULL))
confint(pairs(Semamod_lsmobj),by=NULL)
```

## 5.2 Ap\_Trypsin-like analysis


```
Trypsinmod<-lm(Trypsin~oral_disc+Symbiont*Time, data = qPCR)
anova(Trypsinmod)

Trypsinmod_lsmobj <- lsmeans(Trypsinmod, ~ Symbiont|Time)
summary(as.glht(pairs(Trypsinmod_lsmobj),by=NULL))
confint(pairs(Trypsinmod_lsmobj),by=NULL)
```

LS0tCnRpdGxlOiAiU3RhdGlzdGljYWwgYW5hbHlzZXMiCm91dHB1dDoKICBodG1sX25vdGVib29rOiBkZWZhdWx0CiAgaHRtbF9kb2N1bWVudDogZGVmYXVsdAogIHBkZl9kb2N1bWVudDogZGVmYXVsdAotLS0KCgpgYGB7ciwgZWNobz1GQUxTRX0Ka25pdHI6Om9wdHNfY2h1bmskc2V0KHdhcm5pbmcgPSBGLCBtZXNzYWdlID0gRikKYGBgCgpgYGB7ciwgZWNobz1GQUxTRX0KIyByZXByb2R1Y2liaWxpdHkKcmVxdWlyZShjaGVja3BvaW50KQpjaGVja3BvaW50KCcyMDE3LTAzLTIzJykKCiMgYnVpbGRpbmcgdGhlIGRvY3VtZW50CnJlcXVpcmUoa25pdHIpCnJlcXVpcmUocm1hcmtkb3duKQpyZXF1aXJlKGZvcm1hdFIpCgojIGRhdGEgYW5kIHN0YXRpc3RpY3MgCnJlcXVpcmUobmxtZSkKcmVxdWlyZShsc21lYW5zKQpyZXF1aXJlKG11bHRjb21wKQpyZXF1aXJlKGRwbHlyKQoKIyBwbG90dGluZwpyZXF1aXJlKGxhdHRpY2UpCnJlcXVpcmUoY293cGxvdCkKCgpgYGAKCiMgMS4gVFNQIGFudGlib2R5IGV4cGVyaW1lbnQKCiMjIDEuMSBUU1AgYW50aWJvZHkgbW9kZWwKYGBge3IgVFNQX2FudGlib2R5X21vZGVsfQoKYW50aVRTUF9tb2RfZGF0YSA8LSByZWFkLmNzdignRmlndXJlIDVBIHNvdXJjZSBkYXRhMSBtb2RlbC5jc3YnLCBzdHJpbmdzQXNGYWN0b3JzID0gRikgJT4lIAogIG11dGF0ZShJbmZlY3Rpb24gPSBpbmZlY3Rpb24rMC4wMSwKICAgICAgIGFuZW1vbmUgPSBwYXN0ZShBbmVtb25lLGhvdXJzLFRyZWF0bWVudCksCiAgICAgICBIb3VycyA9IGFzLmZhY3Rvcihob3VycykpIAoKIyBydW4gbWl4ZWQgZWZmZWN0cyBtb2RlbCAtIG5sbWUgZm9yIGNvbnRyYXN0IG1ldGhvZHMKYW50aVRTUF9tb2RlbF90ZXN0IDwtIGxtZShsb2coSW5mZWN0aW9uKX4gSG91cnMqVHJlYXRtZW50LAogICAgICAgICAgICAgICAgICAgICAgICAgIHJhbmRvbT1+MXxhbmVtb25lLAogICAgICAgICAgICAgICAgICAgICAgICAgIGRhdGE9YW50aVRTUF9tb2RfZGF0YSkKCnBsb3QoYW50aVRTUF9tb2RlbF90ZXN0KQoKYGBgCgpgYGB7ciBzdW1tYXJpZXNfVFNQfQoKc21fdGVzdCA8LSBzdW1tYXJ5KGFudGlUU1BfbW9kZWxfdGVzdCkKYW5vdmEoYW50aVRTUF9tb2RlbF90ZXN0LCB0eXBlPSdtJykKYGBgCgojIyAxLjIgVFNQIGFudGlib2R5IG1vZGVsIGNvbnRyYXN0cyBhdCB0aW1lIHBvaW50cwoKVGhlc2UgYXJlIGNvbnRyYXN0cyBiZXR3ZWVuIHRoZSBGU1cgYW5kIElHRyBjb250cm9scyBhbmQgdGhlIGFudGktVFNSIHRyZWF0bWVudAoKYGBge3IgY29udHJhc3RzX1RTUH0KCmFudGlUU1BfbHNtb2JqIDwtIGxzbWVhbnMoYW50aVRTUF9tb2RlbF90ZXN0LCB+IFRyZWF0bWVudHxIb3VycykKCgpzdW1tYXJ5KGFzLmdsaHQocGFpcnMoYW50aVRTUF9sc21vYmopLGJ5PU5VTEwpKQpjb25maW50KHBhaXJzKGFudGlUU1BfbHNtb2JqKSkKCmBgYAoKIyMgMS4zIFRTUCBhbnRpYm9keSBtb2RlbCBwbG90CgpgYGB7ciBnZW5lcmF0ZV9wbG90X1RTUH0KIyBzdW1tYXJ5IGZpbGUgb2YgbWVhbnMgYW5kIFNFIHBlciB0cmVhdG1lbnQgYW5kIHRpbWUtcG9pbnQKYW50aVRTUCA8LSByZWFkLmNzdignRmlndXJlIDVBIHNvdXJjZSBkYXRhMi5jc3YnLCBzdHJpbmdzQXNGYWN0b3JzID0gRikKCmFudGlUU1AkVHJlYXRtZW50W2FudGlUU1AkVHJlYXRtZW50ID09ICdUU1AgYW50aWJvZHknXSA8LSAnVGhyb21ib3Nwb25kaW4gYW50aWJvZHknCgoKZ2dmMSA8LSBnZ3Bsb3QoYW50aVRTUCxhZXMoeD1Ib3Vycyx5PVguLkluZmVjdGlvbixjb2xvdXIgPSBUcmVhdG1lbnQsIHNoYXBlID0gVHJlYXRtZW50KSkgKyAKICBnZW9tX2xpbmUoKSArIAogIGdlb21fcG9pbnQoKSArCiAgZ2VvbV9wb2ludHJhbmdlKGFlcyh5bWluID0gWC4uSW5mZWN0aW9uIC0gc2UsIHltYXggPSBYLi5JbmZlY3Rpb24gKyBzZSkpICsKICBnZW9tX3RleHQoZGF0YT1hbnRpVFNQLGFlcyh4PUhvdXJzLCB5PVguLkluZmVjdGlvbitzZSswLjUsbGFiZWw9YyhOQSxOQSxOQSxOQSxOQSwnKionLE5BLE5BLCcqKionLE5BLE5BLCcqKionLE5BLE5BLCcqKionKSksc2hvdy5sZWdlbmQgPSBGQUxTRSkrCiAgI3NjYWxlX3lfY29udGludW91cyhsYWJlbHMgPSBwZXJjZW50KSsKICBzY2FsZV94X2NvbnRpbnVvdXMoYnJlYWtzID0gYygwLDI0LDQ4LDcyLDk2LDEyMCkpKwogIHlsYWIoJycpICsKICBzY2FsZV9jb2xvdXJfbWFudWFsKCdUaHJvbWJvc3BvbmRpbiBhbnRpYm9keScsdmFsdWVzID0gYygiIzU2QjRFOSIsIiNFNjlGMDAiLCAgIiMwMDlFNzMiKSkgKwogIHNjYWxlX3NoYXBlX2Rpc2NyZXRlKCdUaHJvbWJvc3BvbmRpbiBhbnRpYm9keScpKwogIHRoZW1lX2Nvd3Bsb3QoKSsgCiAgdGhlbWUobGVnZW5kLnRpdGxlID0gZWxlbWVudF90ZXh0KGNvbG91ciA9IE5BKSwKICAgICAgICBheGlzLnRpdGxlLnggPSBlbGVtZW50X3RleHQoY29sb3VyID0gTkEpLAogICAgICAgIGF4aXMudGV4dC54ID0gZWxlbWVudF90ZXh0KGNvbG91ciA9IE5BKSkKCmBgYAoKIyAyLiBTb2x1YWJsZSBUU1AgZXhwZXJpbWVudAoKIyMgMi4xIFNvbHVhYmxlIFRTUCBtb2RlbAoKYGBge3IgU29sdWFibGVfVFNQX21vZGVsfQoKdHNwX3NvbHVibGVfbW9kX2RhdGEgPC0gcmVhZC5jc3YoJ0ZpZ3VyZSA1QiBzb3VyY2UgZGF0YTEgbW9kZWwuY3N2Jywgc3RyaW5nc0FzRmFjdG9ycyA9IEYpICU+JSAKICBtdXRhdGUoSW5mZWN0aW9uID0gaW5mZWN0aW9uKzAuMDEsCiAgICAgICAgIGFuZW1vbmUgPSBwYXN0ZShBbmVtb25lLGhvdXJzLFRyZWF0bWVudCksCiAgICAgICAgIEhvdXJzID0gYXMuZmFjdG9yKGhvdXJzKSkgCgojIHJ1biBtaXhlZCBlZmZlY3RzIG1vZGVsCnRzcF9zb2x1YmxlX21vZGVsX3Rlc3QgPC0gbG1lKGxvZyhJbmZlY3Rpb24pfiBIb3VycypUcmVhdG1lbnQsCiAgICAgICAgICAgICAgICAgICAgICAgICAgICAgIHJhbmRvbT1+MXxhbmVtb25lLAogICAgICAgICAgICAgICAgICAgICAgICAgICAgICBkYXRhPXRzcF9zb2x1YmxlX21vZF9kYXRhKQoKCnBsb3QodHNwX3NvbHVibGVfbW9kZWxfdGVzdCkKYGBgCgpgYGB7ciBzb2xfVFNQX3N1bW1hcmllc30KIyBzdW1tYXJpZXMKc21fdGVzdCA8LSBzdW1tYXJ5KHRzcF9zb2x1YmxlX21vZGVsX3Rlc3QpCmFub3ZhKHRzcF9zb2x1YmxlX21vZGVsX3Rlc3QsIHR5cGU9J20nKQpgYGAKCiMjIDIuMiBTb2x1YWJsZSBUU1AgbW9kZWwgY29udHJhc3RzCgpgYGB7ciBjb250cmFzdHNfc29sX1RTUH0KCnRzcF9zb2x1YmxlX2xzbW9iaiA8LSBsc21lYW5zKHRzcF9zb2x1YmxlX21vZGVsX3Rlc3QsIH4gVHJlYXRtZW50fEhvdXJzKQoKc3VtbWFyeShhcy5nbGh0KHBhaXJzKHRzcF9zb2x1YmxlX2xzbW9iaiksYnk9TlVMTCkpCmNvbmZpbnQocGFpcnModHNwX3NvbHVibGVfbHNtb2JqKSxieT1OVUxMKQoKYGBgCgojIyAyLjMgU29sdWFibGUgVFNQIHBsb3QKCmBgYHtyIGdlbmVyYXRlX3Bsb3Rfc29sX1RTUH0KIyBzdW1tYXJ5IGZpbGUgb2YgbWVhbnMgYW5kIFNFIHBlciB0cmVhdG1lbnQgYW5kIHRpbWUtcG9pbnQKdHNwX3NvbHVibGUgPC0gcmVhZC5jc3YoJ0ZpZ3VyZSA1QiBzb3VyY2UgZGF0YTIuY3N2Jywgc3RyaW5nc0FzRmFjdG9ycyA9IEYpCgp0c3Bfc29sdWJsZSRUcmVhdG1lbnRbdHNwX3NvbHVibGUkVHJlYXRtZW50ID09ICdIcyBUU1AtMSddIDwtICdIcyBUU1InCgoKZ2dmMiA8LSBnZ3Bsb3QodHNwX3NvbHVibGUsYWVzKHg9SG91cnMsIAogICAgICAgICAgICAgICAgICAgICAgIHk9WC4uSW5mZWN0aW9uLCAKICAgICAgICAgICAgICAgICAgICAgICBjb2xvdXIgPSBUcmVhdG1lbnQsIHNoYXBlID0gVHJlYXRtZW50KSkgKyAKICBnZW9tX2xpbmUoKSArIAogIGdlb21fcG9pbnQoKSArCiAgZ2VvbV9wb2ludHJhbmdlKGFlcyh5bWluID0gWC4uSW5mZWN0aW9uIC0gc2UsIHltYXggPSBYLi5JbmZlY3Rpb24gKyBzZSkpICsKICBnZW9tX3RleHQoZGF0YT10c3Bfc29sdWJsZSxhZXMoeD1Ib3VycywgeT1YLi5JbmZlY3Rpb24rc2UrYyhyZXAoMC41LDUpLDEuNSxyZXAoMC41LDQpKSxsYWJlbD1jKE5BLE5BLE5BLCcqKionLE5BLE5BLE5BLCcqKicsTkEsTkEpKSxzaG93LmxlZ2VuZCA9IEZBTFNFKSsKICAjc2NhbGVfeV9jb250aW51b3VzKGxhYmVscyA9IHBlcmNlbnQpKwogIHNjYWxlX3hfY29udGludW91cyhicmVha3MgPSBjKDAsMjQsNDgsNzIsOTYsMTIwKSkrCiAgeWxhYignJSBjb2xvbmlzYXRpb24nKSArCiAgc2NhbGVfY29sb3VyX21hbnVhbCgnVGhyb21ib3Nwb25kaW4gYW50aWJvZHknLHZhbHVlcyA9IGMoIiM1NkI0RTkiLCIjQ0M3OUE3IikpICsKICBzY2FsZV9zaGFwZV9kaXNjcmV0ZSgnVGhyb21ib3Nwb25kaW4gYW50aWJvZHknKSsKICB0aGVtZV9jb3dwbG90KCkrIAogIHRoZW1lKGxlZ2VuZC50aXRsZSA9IGVsZW1lbnRfdGV4dChjb2xvdXIgPSBOQSksCiAgICAgICAgYXhpcy50aXRsZS54ID0gZWxlbWVudF90ZXh0KGNvbG91ciA9IE5BKSwKICAgICAgICBheGlzLnRleHQueCA9IGVsZW1lbnRfdGV4dChjb2xvdXIgPSBOQSkpCmBgYAoKIyAzIFRTUiBwZXB0aWRlIGNvbG9uaXNhdGlvbgoKIyMgMy4xIFRTUiBwZXB0aWRlIGNvbG9uaXNhdGlvbgoKYGBge3IgcGVwdGlkZV9tb2RlbH0KcGVwdGlkZV9tb2RfZGF0YSA8LSByZWFkLmNzdignRmlndXJlIDVDIHNvdXJjZSBkYXRhMSBtb2RlbC5jc3YnLCBzdHJpbmdzQXNGYWN0b3JzID0gRikgJT4lIAogIG11dGF0ZShJbmZlY3Rpb24gPSBpbmZlY3Rpb24rMC4wMSwKICAgICAgICAgYW5lbW9uZSA9IHBhc3RlKEFuZW1vbmUsaG91cnMsVHJlYXRtZW50KSwKICAgICAgICAgSG91cnMgPSBhcy5mYWN0b3IoaG91cnMpKSAKCnBlcHRpZGVfbW9kX2RhdGEkVHJlYXRtZW50IDwtIGFzLmZhY3RvcihwZXB0aWRlX21vZF9kYXRhJFRyZWF0bWVudCkKcGVwdGlkZV9tb2RfZGF0YSRIb3VycyA8LSBhcy5mYWN0b3IocGVwdGlkZV9tb2RfZGF0YSRIb3VycykKCiMgcnVuIG1peGVkIGVmZmVjdHMgbW9kZWwKcGVwdGlkZV9tb2RlbF90ZXN0IDwtIGxtZShsb2coSW5mZWN0aW9uKX4gSG91cnMqVHJlYXRtZW50LAogICAgICAgICAgICAgICAgICAgICByYW5kb209fjF8YW5lbW9uZSwKICAgICAgICAgICAgICAgICAgICAgIGRhdGE9cGVwdGlkZV9tb2RfZGF0YSkKCnBsb3QocGVwdGlkZV9tb2RlbF90ZXN0KQpgYGAKCmBgYHtyIHN1bW1hcmllc30Kc21fdGVzdCA8LSBzdW1tYXJ5KHBlcHRpZGVfbW9kZWxfdGVzdCkKYW5vdmEocGVwdGlkZV9tb2RlbF90ZXN0LHR5cGU9J21hcmdpbmFsJykKYGBgCgojIyAzLjIgUGVwdGlkZSBjb250cmFzdHMKCmBgYHtyIHBlcHRpZGVfY29udHJhc3RzfQogICAgICAgICAgICAgICAgICAgCnBlcHRpZGVfbHNtb2JqIDwtIGxzbWVhbnMocGVwdGlkZV9tb2RlbF90ZXN0LCB+IFRyZWF0bWVudHxIb3VycykKCnN1bW1hcnkoYXMuZ2xodChwYWlycyhwZXB0aWRlX2xzbW9iaiksYnk9TlVMTCkpCmNvbmZpbnQocGFpcnMocGVwdGlkZV9sc21vYmopLCBieT1OVUxMKQogICAgICAgICAgICAgICAgICAgCmBgYAoKIyMgMy4zIFBsb3QgcGVwdGlkZSBjb2xvbmlzYXRpb24KCmBgYHtyIHBsb3RfcGVwdGlkZXN9CnBlcHRpZGVfaW5mZWN0aW9uIDwtIHJlYWQuY3N2KCdGaWd1cmUgNUMgc291cmNlIGRhdGEyLmNzdicpCgpnZ2YzIDwtIGdncGxvdChwZXB0aWRlX2luZmVjdGlvbixhZXMoeD1Ib3Vycyx5PVguLkluZmVjdGlvbixjb2xvdXIgPSBUcmVhdG1lbnQsIHNoYXBlID0gVHJlYXRtZW50KSkgKyAKICBnZW9tX2xpbmUoKSArIAogIGdlb21fcG9pbnQoKSArCiAgZ2VvbV9wb2ludHJhbmdlKGFlcyh5bWluID0gWC4uSW5mZWN0aW9uIC0gc2UsIHltYXggPSBYLi5JbmZlY3Rpb24gKyBzZSkpICsKICBnZW9tX3RleHQoZGF0YT1wZXB0aWRlX2luZmVjdGlvbixhZXMoeD1Ib3VycywgeT1YLi5JbmZlY3Rpb24rc2UrMC41LGxhYmVsPWMoTkEsTkEsTkEsTkEsTkEsJyoqKicsTkEsTkEsJyoqKicsTkEsTkEsJyoqKicsTkEsTkEsTkEpKSxzaG93LmxlZ2VuZCA9IEZBTFNFKSsKICBnZW9tX3RleHQoZGF0YT1wZXB0aWRlX2luZmVjdGlvbixhZXMoeD1Ib3VycywgeT1YLi5JbmZlY3Rpb24tc2UtYyhyZXAoMSxucm93KHBlcHRpZGVfaW5mZWN0aW9uKS0yKSwwLjEsTkEpLGxhYmVsPWMoTkEsTkEsTkEsTkEsJyoqKicsTkEsTkEsJyoqKicsTkEsTkEsJyoqKicsTkEsTkEsTkEsTkEpKSxudWRnZV95ID0gLTAuMixzaG93LmxlZ2VuZCA9IEZBTFNFKSsKICAjc2NhbGVfeV9jb250aW51b3VzKGxhYmVscyA9IHBlcmNlbnQpKwogIHNjYWxlX3hfY29udGludW91cyhicmVha3MgPSBjKDI0LDQ4LDcyLDk2LDEyMCkpKwogIHlsYWIoJycpICsKICBzY2FsZV9jb2xvdXJfbWFudWFsKCdUaHJvbWJvc3BvbmRpbiBhbnRpYm9keScsdmFsdWVzID0gYygiIzU2QjRFOSIsICIjMDA3MkIyIiwgIiNENTVFMDAiKSkgKwogIHNjYWxlX3NoYXBlX2Rpc2NyZXRlKCdUaHJvbWJvc3BvbmRpbiBhbnRpYm9keScpKwogIHRoZW1lX2Nvd3Bsb3QoKSsgCiAgdGhlbWUobGVnZW5kLnRpdGxlID0gZWxlbWVudF90ZXh0KGNvbG91ciA9IE5BKSkKCmBgYAoKIyA0LiBQbG90cwoKYGBge3IgcGxvdHN9CmFsbF9maWd1cmVzIDwtIGNvd3Bsb3Q6OnBsb3RfZ3JpZChnZ2YxLGdnZjIsZ2dmMyxuY29sPTEpCmFsbF9maWd1cmVzCmdnc2F2ZShhbGxfZmlndXJlcyxmaWxlbmFtZSA9ICdGaWd1cmUgNS5wZGYnLHdpZHRoID0gNixoZWlnaHQ9OCkKYGBgCgojIDUuIHFQQ1IgYW5hbHlzaXMKCmBgYHtyfQpxUENSIDwtIHJlYWQuY3N2KCdGaWd1cmUgNiBzb3VyY2UgZGF0YS5jc3YnLAogICAgICAgICAgICAgICAgIHN0cmluZ3NBc0ZhY3RvcnMgPSBGKQoKcVBDUiRUaW1lIDwtIGFzLmZhY3RvcihxUENSJFRpbWUpCnFQQ1IkU3ltYmlvbnQgPC0gYXMuZmFjdG9yKHFQQ1IkU3ltYmlvbnQpCgoKYGBgCgojIyA1LjEgQXBfU2VtYTUgYW5hbHlzaXMKCmBgYHtyIFNlbWF9ClNlbWFtb2Q8LWxtKFNlbWF+b3JhbF9kaXNjK1N5bWJpb250KlRpbWUsIGRhdGEgPSBxUENSKQphbm92YShTZW1hbW9kKQoKU2VtYW1vZF9sc21vYmogPC0gbHNtZWFucyhTZW1hbW9kLCB+IFN5bWJpb250fFRpbWUpCnN1bW1hcnkoYXMuZ2xodChwYWlycyhTZW1hbW9kX2xzbW9iaiksYnk9TlVMTCkpCmNvbmZpbnQocGFpcnMoU2VtYW1vZF9sc21vYmopLGJ5PU5VTEwpCgpgYGAKCiMjIDUuMiBBcF9Ucnlwc2luLWxpa2UgYW5hbHlzaXMKCmBgYHtyIFRyeXBzaW59CgpUcnlwc2lubW9kPC1sbShUcnlwc2lufm9yYWxfZGlzYytTeW1iaW9udCpUaW1lLCBkYXRhID0gcVBDUikKYW5vdmEoVHJ5cHNpbm1vZCkKClRyeXBzaW5tb2RfbHNtb2JqIDwtIGxzbWVhbnMoVHJ5cHNpbm1vZCwgfiBTeW1iaW9udHxUaW1lKQpzdW1tYXJ5KGFzLmdsaHQocGFpcnMoVHJ5cHNpbm1vZF9sc21vYmopLGJ5PU5VTEwpKQpjb25maW50KHBhaXJzKFRyeXBzaW5tb2RfbHNtb2JqKSxieT1OVUxMKQoKYGBgCg==
